# Changes in weight, physical activity, sedentary behaviour and dietary intake during the transition to higher education: a prospective study

**DOI:** 10.1186/s12966-015-0173-9

**Published:** 2015-02-15

**Authors:** Benedicte Deforche, Delfien Van Dyck, Tom Deliens, Ilse De Bourdeaudhuij

**Affiliations:** Department of Public Health, Ghent University, De Pintelaan 185, 9000 Ghent, Belgium; Department of Movement and Sports Sciences, Ghent University, Watersportlaan 2, 9000 Ghent, Belgium; Research Foundation Flanders (FWO), Egmontstraat 5, 1000 Brussels, Belgium; Department of Human Biometrics and Biomechanics, Vrije Universiteit Brussel, Pleinstraat 2, 1050 Brussels, Belgium

**Keywords:** Body mass index, Freshman 15, Health behaviour, Longitudinal study, Students, Body weight changes

## Abstract

**Background:**

The transition to higher education involves a significant life change and might be accompanied with less healthy behaviours. However, the only longitudinal study that spanned the period from high school to college/university was limited to self-reported weight. Other studies assessed objective weight, but only at the start of the first semester at college/university and used retrospective questionnaires to asses health behaviours in high school. This study investigated changes in objectively assessed weight and prospective health behaviours during the transition from high school to college/university in Belgian students and examined which health behaviour changes were related to weight change.

**Methods:**

A sample of 291 students was followed from the final year of high school until the second year of college/university. Body mass index (BMI) and waist circumference were measured objectively. Physical activity, sedentary behaviours and dietary intake were estimated using validated questionnaires. In order to study changes in BMI and health behaviours, 2 × 2 (time × gender) Repeated Measures ANOVA analyses were conducted. A stepwise multiple regression analysis was executed to investigate the association between changes in health behaviours and BMI changes, and the moderating effect of gender.

**Results:**

On average students gained 2.7 kg with a greater increase in boys (boys: 4.2 kg, girls: 1.9 kg). Active transportation and sport participation decreased. Some sedentary behaviours (watching TV/DVD, playing computer games) decreased, while others (internet use, studying) increased. Consumption of different foods decreased, while alcohol consumption increased. A higher decrease in sport participation, a higher increase in internet use and a lower increase in studying were related to a greater increase in BMI. An increase in alcohol consumption only contributed to weight gain in boys, whereas a decrease in fruit/vegetable intake only contributed to weight gain in girls.

**Conclusion:**

We can conclude that the transition to higher education is an at risk period for weight gain and unfavourable changes in health behaviours. Interventions to prevent weight gain in college/university students should therefore already start in high school with a somewhat different focus in boys versus girls.

## Introduction

In order to prevent the rising prevalence of overweight it is important to detect at risk periods of weight gain and development of unhealthy behaviours [1]. The transition from high school to college or university has been recognized as such an at risk period [2-4]. This transition to higher education involves a significant life change for many students as they often start living away from their parental home for the first time, get more freedom and make new friends [5-8]. This transition might be accompanied with abandonment of routines and habits established at high school and adoption of a new lifestyle. The purpose of this paper is to study changes in weight and health behaviours during this transition period and to investigate which health behaviours are associated with weight gain in college or university students.

Prior work in this field shows several shortcomings. Longitudinal studies that span the period from high school to college or university are scarce. Only one US study assessed students in their senior year of high school for baseline measurements, but only self-reported weight was assessed and data were collected in 1995–1996, almost twenty years ago [9]. To the best of our knowledge, all studies investigating objectively measured weight gain in students are limited to data collected when students are already at college or university. Evidence from these studies shows that European (Belgian) freshman students gain on average 1 kg during the first semester [10], whereas US and Canadian freshman students showed larger weight gains (ranging from 0.9 to 3.1 kg) [2,4]. This could be explained by possible lifestyle and socio-cultural differences between North-American and European students (e.g. stronger fast food culture in US and shorter distance between home and student residence in Europe). Some studies [10-14] found a greater increase in body mass in boys versus girls, while other studies [15-19] found no gender differences in weight gain. No previous European studies have investigated weight changes beyond the first semester at college/university.

Some previous studies documented unhealthy behaviours among college or university students including decreased physical activity, poor dietary quality, increased alcohol consumption and decreased sleep duration [14,20,21]. However the only studies that examined changes in behaviours that occur as students make the transition from high school to college or university (as opposed to during time spent at college or university), either used a retrospective questionnaire [21,22] or did baseline assessments during a freshman orientation course two weeks before the start of the first semester [23].

Several studies investigated associations between changes in health behaviours and weight gain in college or university [2,4]. While some studies found that decreased physical activity or low physical activity levels were related to weight gain [18,24,25], other studies found no differences in weight gain according to physical activity [10,26]. Only one previous study investigated sedentary behaviour as a potential contributor to weight gain in university students. It was found that students watching more TV or DVDs during weekdays showed higher weight gain [10]. Some studies found decreased consumption of fruit and vegetables and increased consumption of unhealthy food items (e.g. high-fat foods, soft-drinks, sweet snacks/desserts) to be associated with freshmen’s weight gain [26,27], while other studies found no associations between dietary intake and weight gain [9,18,25]. Despite mixed findings of previous studies, following health behaviours were identified as potential contributors of weight gain in college or university students: a decrease in physical activity, higher amount of TV/DVD watching, unhealthy eating behaviours.

Only two studies examined health behavioural correlates of weight gain by gender. The first study found that weight gain in college students was related to increased alcohol consumption in boys and lower fruit/vegetable consumption at baseline in girls [28]. In the second study, boys who reported high levels of physical activity as well as high intensity levels were found to gain weight, which suggests that the weight gain may have been the result of an increase in lean body mass [9].

In summary, while previous studies have provided important information, there is a lack of studies using objective assessment of weight and including baseline measures at the end of high school. Studies including the transition period from high school to college or university are needed in order to determine behaviour changes associated with weight gain in college or university students. When collecting baseline measurements at the start of the freshman year, some health behaviours may already have changed and important changes during the transition might be missed.

Therefore the first purpose of this study was to investigate changes in objectively measured weight/body composition and self-reported health behaviours (physical activity, sedentary behaviour and dietary intake) during the transition from high school to college or university in Belgian students. The second purpose was to determine which hypothesized health behaviour changes (decreased physical activity, increased sedentary behaviour and less healthy eating behaviour) during the transition to higher education were related to weight change. Furthermore, the moderating effect of gender was investigated with regard to both research questions.

## Methods

### Participants and protocol

To recruit participants, 150 randomly selected mixed gender public high schools providing general (= academic) education in East- and West-Flanders, two regions in Belgium, were contacted by phone, of which 48 agreed to participate. All last year students (n = 2726, 17.3 ± 0.5 yrs) were measured at school during class hours in the last semester of high school (wave 1 in February-March 2008; wave 2 in February-March 2009). In order to be able to recontact these students 1.5 years later, each student was asked to provide three different phone numbers. All participating students were recontacted 1.5 years after baseline measurements, at the start of the second year at college or univerisity (wave 1 in October 2009; wave 2 in October 2010). Every student was phoned a maximum of three times on each available phone number on different days and times of the day. For practical reasons, only students studying at a college or university in one selected city (Ghent, location of research group) were eligible to participate in the follow-up measurements. Of the total sample tested during the last year of high school 26.9% could not be reached at follow-up, 19.8% studied in another city, 1.5% were not studying and 0.1% had passed away. From the 1407 participants studying in Ghent at follow-up, 291 (20.7%) participated in the follow-up measurements in the second month of the second year of college or university, whereas 1116 students (79.3%) did not participate further.

The final sample consisted of 291 participants (33.3% boys). There were no differences in gender (p = 0.5) and body mass index (BMI) (p = 0.2) between participants (33.3% boys; BMI: 21.2 ± 2.4 kg/m^2^) and drop-outs (35.9% boys; BMI: 21.4 ± 2.9 kg/m^2^).

Participating students were invited to a university research room of their choice (at different locations in the city) for data collection. At follow-up, participating students received an incentive (movie ticket). The study protocol was approved by the ethical committee of the University Hospital. Informed consent was obtained from school directors, parents and students before the start of the study.

### Measurements

#### Anthropometric measurements

Weight, height and waist circumference were objectively measured according to international standards [29]. Height was measured in high school to the nearest 0.1 cm using a portable stadiometer (Seca 214, Hamburg, Germany). Body mass was measured at both time points to the nearest 0.1 kg on a digital balance scale (Seca 813 Robusta, max 200 kg, Hamburg, Germany) with the subject wearing lightweight clothing and no shoes. BMI was calculated as weight (kg)/height^2^ (m^2^) [30]. Waist circumference was measured at both time points to the nearest 0.1 cm at the narrowest part of the waist, between the lower costal (10th rib) border and the iliac crest [29] using a standard anthropometric tape (Seca 200, Hamburg, Germany).

#### Demographic variables

Self-reported demographic variables measured at both time points included gender, birth date, smoking status and parental educational level. At follow-up, residency (living at home, living in a student residence) was assessed.

#### Physical activity

At both time points, activity levels were determined using the Flemish Physical Activity Questionnaire (FPAQ), which was found to be a reliable and reasonably valid questionnaire for the assessment of different dimensions of physical activity in adolescents [31]. The test-retest intraclass correlation coefficients exceeded .70 and pearson correlations with accelerometer data ranged between .43 and .48. To assess “active transportation”, minutes/week spent in active transport (walking, cycling, using step/skate/rollerblades) to school/college/university (week days only) and in leisure-time (week and weekend days apart) were added up. “Sport participation” was assessed by adding minutes/week spent in sports at school/college/university (week days only) and in leisure-time sports (week and weekend days apart).

#### Sedentary behaviour

Sedentary behaviour was measured at both time points using the Sedentary Behaviour Questionnaire (SBQ) which was previously validated in adults [32]. Intraclass correlation coefficients were acceptable for all 9 items (range = .51–.93). The SBQ items were also found to be related with self-reported sitting time (assessed by International Physical Activity Questionnaire), accelerometer inactivity minutes and BMI. Furthermore, the SBQ showed good construct validity in adolescents [33].

Sedentary behaviour was measured in minutes per typical day spent on various sedentary behaviours (TV/DVD viewing, playing computer games, internet use, studying (writing, reading, computer use), playing video games, sitting and listening to music, sitting while phoning or texting, sitting to hang out or talk with friends and family, reading a book or magazine (not for school), doing inactive hobbies, sitting in motorized transport) [32,33]. The questions were asked for a usual weekday and weekend day separately. Response options were: none, 15 minutes, 30 minutes, 1 hour, 2 hours, 3 hours, or 4 or more hours. Responses for usual weekday and weekend day separately were multiplied by the appropriate days per week (5 for weekday and 2 for weekend day), summed and divided by 7 to obtain the final score for each sedentary behaviour in minutes per day.

#### Dietary intake

Dietary intake was assessed at both time points using the Food Frequency Questionnaire (FFQ) from the Health Behavior in School aged Children study [34]. This FFQ showed good reliability in 13–14 year old children (spearman correlations ranged from .57 to .78) and good relative validity (compared to a 24-hour food behaviour checklist) in 11–18 year olds [35].

The FFQ asked about the consumption frequency of important sources of dietary fibre (fruit, vegetables, breakfast cereals (for example cornflakes, choco pops, muesli…), white bread, brown bread) and calcium ((semi-)skimmed milk, whole fat milk, cheese, other milk products (for example yogurt, quark, chocolate milk, pudding… )) and items typical for youth food culture (crisps or chips, sweets or chocolates, carbonated sugared soft drinks, diet soft drinks, alcohol beverages). The response categories for each food item were: ‘never’, ‘less than once a week’, ‘once a week’, ‘2–4 days/week’, ‘5–6 days/week’, ‘once a day, every day’ and ‘every day, more than once’. These response categories were recoded as follows: ‘never’ = 0, ‘less than once a week’ = 0.25 (reflecting a consumption frequency of once every four weeks), ‘once a week’ = 1, ‘2–4 days a week’ = 3 (midpoint of the interval), ‘5–6 days a week’ = 5.5 (midpoint of the interval) and ‘once a day, every day’ = 7 and ‘more than once a day, every day’ = 14.

From this Food Frequency Questionnaire different indices were calculated [33]. For the ‘Fruit and Vegetables Index’, the consumption frequencies of fruit and vegetables were summed. For the ‘Fiber Index’, the consumption frequencies of fruit, vegetables and brown bread were cumulated. For the ‘Calcium-Index’, the consumption frequencies of whole fat milk, semi-skimmed milk, cheese and other milk products were summed. In order to have a balanced diet, diversity or variety of items from different food groups is advocated. Therefore a ‘variety index’ was composed by summing the consumption frequencies of fruits, vegetables, brown bread, whole fat milk, semi-skimmed milk, cheese and other milk products. Finally the consumption frequency of carbonated sugared soft drinks, sweets, chips and crisps, 4 popular food items of low nutritional value were cumulated to form an ‘Excess Index’.

In addition to the HBSC FFQ, the absolute number of beers or other alcoholic consumptions per week was assessed at both time points.

### Statistical analyses

Data were analyzed using SPSS Statistics 21.0. In order to study changes in body composition and health behaviours between the final year of high school (baseline) and the second year of college/university (follow-up) in boys and girls, 2 × 2 (time × gender) Repeated Measures ANOVA analyses were conducted. In case of interaction effects, paired samples t-tests were performed for boys and girls separately.

A stepwise multiple regression analysis was executed to investigate the association between changes in health behaviours and BMI changes, and the moderating effect of gender. Before running this analysis, residualized change scores for BMI and health behaviours were created by regressing the follow-up values onto their respective baseline values. The residualized change scores can be interpreted as the amount of change in BMI and health behaviours between baseline and follow-up, independent of baseline levels and are preferable to simple change scores because they eliminate autocorrelated error and regression to the mean effects [36,37].

Before building the final model, associations with BMI change were tested individually for all health behaviours/cross-product terms (gender × health behaviours). Only those variables that showed an association of r > 0.10 with BMI change were selected to be entered in the final model. Next, intercorrelations were computed between all selected independent variables. For variables showing intercorrelations higher than 0.60, only the variable with the highest bivariate correlation with the dependent variable (BMI change) was kept, the others were removed from the model to reduce multicollinearity.

In a first step, the socio-demographic covariates (gender, maternal educational level as a proxy for socio-economic status and residency) were entered. In a second step, the health behavioural variables were entered as independent variables. In the third step, the cross-product terms of gender and health behaviours were entered in the model to examine the moderating effects of gender. In case of interactions, separate regression models (boys versus girls) were run to interpret the direction of the interactions.

Effects sizes were reported in the form of Cohen’s d statistic (small = 0.20 – 0.49, medium = 0.50 – 0.79, large ≥ 0.80) [38]. For interpreting main effects 95% confidence intervals were used, while 90% confidence intervals were used for interpreting interaction effects [39].

## Results

### Demographic characteristics

At baseline, mean age was 17.2 ± 0.5 years, 6.9% were smokers. In the total sample, 27.4% of mothers and 33.5% of fathers had less than a college or university/graduate degree, 56.9% of mothers and 36% of fathers had a college degree and 15.7% of mothers and 30.6% of fathers had a university/graduate degree. At follow-up, 70.1% lived in a student residence and 29.9% lived at home with (one of) their parents.

### Changes in body composition and health behaviours

On average students gained 2.7 kg, with boys gaining 4.2 kg (95% CI: 3.4, 5.0, p < 0.001, d = 1.1) and girls 1.9 kg (95% CI: 1.4, 2.5, p < 0.001, d = 0.50). There was a large increase in BMI, with a greater increase in boys (95% CI: 1.1, 1.7, p < 0.001, d = 1.0) versus girls (95% CI: 0.4, 0.8, p < 0.001, d = 0.50), whereas waist circumference only slightly increased in boys (95% CI: 0.6, 3.1, p = 0.005, d = 0.30) and did not change in girls (Table 1). An increase in BMI was observed in 78% of students. At baseline, 5.3% were underweight, 88.7% were normal-weight and 6% were overweight. At follow-up, there was a decrease in number of underweight and normal-weight students (to respectively 3.9% and 84%), but the number of overweight students doubled to 12%.

**Table 1:** **Changes in anthropometrics, physical activity, sedentary behaviour and dietary intake during transition to higher education**

	**Boys (n = 97)**		**Girls (n = 194 )**		**F**_**timexgender df = (1, 289)**_	**p**_**timexgender**_	**F**_**time df = (1, 289)**_	**p**_**time**_
	**High school**	**College/university**	**High school**	**College/university**				
**Anthropometrics**								
Weight (kg)	68.5 ± 8.5	72.7 ± 9.9	59.8 ± 7.4	61.7 ± 8.1	20.8	<0.001	156.5	<0.001
BMI (kg/m^2^)	21.1 ± 2.5	22.6 ± 3.1	21.3 ± 2.4	21.9 ± 2.5	22.6	<0.001	145.3	<0.001
Waist circumference (cm)	76.9 ± 7.0	78.7 ± 7.6	72.8 ± 8.2	72.3 ± 5.6	7.8	0.006	2.6	0.11
**Physical activity (min/week)**								
Active transportation	337 ± 217	224 ± 141	287 ± 188	212 ± 124	2.0	0.154	48.8	<0.001
Sport participation	241 ± 239	138 ± 183	158 ± 201	98 ± 137	3.5	0.062	50.2	<0.001
**Sedentary behaviour (min/day)** ^**1**^								
TV/DVD viewing	88.9 ± 49.2	72.6 ± 45.8	86.9 ± 54.3	77.6 ± 47.8	1.0	0.31	13.9	<0.001
Playing computer/video games	32.2 ± 45.6	23.8 ± 41.6	4.4 ± 15.8	3.0 ± 12.4	4.8	0.03	9.5	0.002
Internet use (not for school)	66.8 ± 48.6	89.8 ± 56.1	78.3 ± 57.2	91.9 ± 60.1	1.6	0.21	24.6	<0.001
Studying (writing, reading, computer use)	84.2 ± 54.9	114.5 ± 65.6	123.0 ± 55.5	144.0 ± 56.4	1.1	0.29	34.3	<0.001
Sitting while listening to music	54.1 ± 64.7	63.9 ± 71.3	59.8 ± 67.0	54.6 ± 70.4	2.3	0.12	0.2	0.63
Sitting while phoning or texting	19.5 ± 29.3	26.3 ± 31.5	35.5 ± 43.0	33.6 ± 44.0	1.9	0.17	0.6	0.44
Sitting to hang out or talk with friends or family	69.2 ± 54.7	84.8 ± 61.3	91.1 ± 62.9	99.4 ± 62.6	0.6	0.43	6.8	0.01
Reading book or magazine (not for school)	22.4 ± 28.9	21.2 ± 29.3	21.2 ± 21.9	21.9 ± 25.0	0.34	0.56	0.04	0.85
Doing inactive hobbies	29.9 ± 39.7	26.2 ± 36.9	34.1 ± 46.7	20.6 ± 36.9	2.5	0.11	7.9	0.005
Sitting in motorized transport	21.2 ± 17.0	17.4 ± 20.7	25.7 ± 28.6	19.9 ± 19.9	0.4	0.53	8.4	0.004
**Dietary intake (consumptions/week)**								
Fruits and vegetables index^2^	17.8 ± 7.8	14.1 ± 8.0	21.7 ± 7.0	18.6 ± 8.3	0.4	0.534	45.2	<0.001
Fiber index^3^	24.9 ± 10.4	19,5 ± 10.6	30.7 ± 10.2	25.8 ± 11.0	0.2	0.665	62,4	<0.001
Calcium index^4^	18.1 ± 10.8	12.8 ± 8.4	21.2 ± 11.3	16.5 ± 11.0	0.1	0.702	54.7	<0.001
Variety index^5^	43.0 ± 17.3	32.3 ± 15.9	51.9 ± 16.7	42.3 ± 17.7	0.3	0.591	101.9	<0.001
Excess index^6^	16.9 ± 9.5	13.7 ± 8.5	13.3 ± 8.4	10.7 ± 8.2	0.3	0.575	28.4	<0.001
Alcohol	4.9 ± 4.9	10.3 ± 10.4	3.0 ± 3.4	4.5 ± 4.1	35.0	<0.001	106.8	<0.001

^1^Responses for weekday and weekend day were multiplied by the appropriate days per week (5 for weekday and 2 for weekend day) and summed to obtain the final score for each sedentary behaviour in hours per week.

^2^summation of consumption frequencies of fruit and vegetables.

^3^summation of consumption frequencies of fruit, vegetables and brown bread.

^4^summation of consumption frequencies of whole fat milk, semi-skimmed milk, cheese and other milk products.

^5^summation of consumption frequencies of fruits, vegetables, brown bread, whole fat milk, semi-skimmed milk, cheese and other milk products.

^6^summation of consumption frequency of carbonated sugared soft drinks, sweets, chips and crisps (4 popular food items of low nutritional value).

Changes in health behaviours are presented in Table 1. Both boys and girls showed a small decrease in sport participation and active transportation, with a somewhat larger decrease in sport participation in boys (95% CI: −148, −59, p < 0.001, d = 0.47) versus girls (95% CI: −84, −37, p < 0.001, d = 0.37).

In both boys and girls, there was a small decrease in TV/DVD viewing (95% CI: −17.8, −5.3, p < 0.001, d = 0.21), inactive hobbies (95% CI: −16.0, −4.6, p < 0.001, d = 0.21), sitting in motorized transport (95% CI: −8.2, −2.1, p = 0.001, d = 0.19), while there was a small increase in internet use (95% CI: 9.9, 23.5, p < 0.001, d = 0.28) and studying (95% CI: 16.0, 32.2, p < 0.001, d = 0.34), a negligible increase in sitting to hang out/talk with friends/family (95% CI: 2.2, 19.2, p = 0.014, d = 0.15) and no change in sitting while listening to music, sitting while phoning/texting and reading. Playing computer/video games slightly decreased only in boys (95% CI: −16, −0.8, p = 0.03, d = 0.22). There was an overall moderate decrease in intake for all different food indices (fruits and vegetables index: 95% CI: −4.3, −2.4, p < 0.001, d = 0.41, fiber index: 95% CI: −6.2, −3.8, p < 0.001, d = 0.48, calcium index: 95% CI: −6.2, −3.7, p < 0.001, d = 0.45, variety index: 95% CI: −11.8, −8.1, p < 0.001, d = 0.63, excess index: 95% CI: −3.8, −1.8, p < 0.001, d = 0.32) in both genders and a somewhat higher increase in alcohol consumption in boys (95% CI: 3.8, 7.0, p < 0.001, d = 0.68) versus girls (95% CI: 1.0, 1.9, p < 0.001, d = 0.45).

### Associations of changes in health behaviours with BMI change

Bivariate correlations higher than 0.10 with BMI change were found for changes in ‘sport participation’, ‘internet use’, ‘studying’, ‘fruit and vegetable intake’, ‘fiber intake’ and ‘alcohol consumption’. As the intercorrelation between ‘fruit and vegetable intake’ and ‘fiber intake’ was high (r = 0.87), only the factor ‘fruit and vegetable intake’ was included in the multiple regression analysis.

Results of the stepwise multiple regression analysis are shown in Table 2 (step 3). Gender was associated with BMI change, with girls having a lower increase in BMI of 0.40 kg/m^2^ compared to boys. A decrease of 1 min/week of sport participation and an increase of 1 min/week of internet use for leisure was related with respectively an increase in BMI of 0.16 and 0.13 kg/m^2^. An increase of 1 min/week in time spent studying was associated with a decrease in BMI of 0.13 kg/m^2^. There was a moderating effect of gender for ‘change in fruit and vegetable intake’ (b = −0.06, 90% CI: −0.12, −0.003, d = 0.22, p = 0.08) and ‘change in alcohol consumption’ (b = 0.08, 90% CI: 0.004, 0.16, d = 0.22, p = 0.08). An increase of 1 consumption/week of fruit and vegetables was associated with a decrease in BMI of 0.13 kg/m^2^ in girls (b = −0.13, 95% CI: −0.26, 0.002, d = 0.24, p = 0.05), but not in boys. On the other hand, an increase of 1 alcohol consumption/week was related with an increase in BMI of 0.30 kg/m^2^ in boys (b = 0.30, 95% CI: 0.09, 0.51, d = 0.36, p = 0.005), but not in girls. Overall, effect sizes were small (Cohen’s d between 0.24 and 0.49). The final regression model explained 12% of the variance in BMI change.

**Table 2:** **Stepwise multiple regression analysis predicting BMI change from gender, health behaviour changes and interaction between gender and health behaviours**

**Step**	**Independent variables**	**Beta (95% CI)**	**p-value**	**d**	**ΔR** ^**2**^ **F(df), p**	**Adj R** ^**2**^ **F(df), p**
1	Gender^1^	−0.57 (−0.82 -0.31)	<0.001	0.57	0.07	0.06
	Residency^2^	−0.07 (−0.34, 0,21)	0.62	0.06	F(4, 286) = 5.1, p = 0.001	F(4,286) = 5.1, p = 0.001
	Socio-economic status^3^	−0.07 (−0.34, 0.21)	0.63	0.06		
	Smoking status^4^	−0.13 (−0.67, 0.4)	0.62	0.06		
2	Gender^1^	−0.49 (−0.74, −0.24)	<0.001	0.49	0.06	0.11
	Residency^2^	−0.14 (−0.41, 0.13)	0.30	0.13	F(5, 281) = 5.3, p = 0.002	F (9, 281) = 5.3, p < 0.001
	Socio-economic status^3^	−0.02 (−0.28, 0.25)	0.89	0.02		
	Smoking status^4^	−0.18 (−0.70, 0.35)	0.51	0.08		
	Change in sport participation	−0.15 (−0.27, −0.03)	0.02	0.31		
	Change in internet use	0.14 (0.02, 0.26)	0.02	0.29		
	Change in time spent studying	−0.14 (−0.26, −0.01)	0.03	0.27		
	Change in fruit and vegetable intake	−0.10 (−0.22, 0.03)	0.13	0.19		
	Change in alcohol consumption	0.07 (−0.05, 0.20)	0.25	0.14		
3	Gender^1^	−0,40 (−0.66, −0.14)	0.003	0.39	0.02	0.12
	Residency^2^	−0,22 (−0.49, 0.06)	0.12	0.20	F(2, 279) = 3.3, p = 0.04	F(9, 279) = 4.9, p < 0.001
	Socio-economic status^3^	0.004 (−0.26, 0.27)	0.98	0.00		
	Smoking status^4^	−0.20 (−0.72, 0.33)	0.46	0.09		
	Change in sport participation	−0.16 (−0.28, −0.04)	0.01	0.32		
	Change in internet use	0.13 (0.01, 0.25)	0.04	0.27		
	Change in time spent studying	−0.13 (−0.25, −0.01)	0.03	0.27		
	Change in fruit and vegetable intake	0.11 (−0.37, 0.60)	0.65	0.06		
	Change in alcohol consumption	−0.21 (−0.61, 0.18)	0.37	0.14		
	Gender × change in fruit and vegetable intake	−0.06 (−0.13, 0.01)	0.08	0.22		
	Gender × change in alcohol consumption	0.08 (−0.01, 0.17)	0.08	0.22		

CI = confidence interval.

^1^ref: boy.

^2^ref: home.

^3^ref: low socio-economic status.

^4^ref: non-smoking.

## Discussion

The purpose of this study was to investigate changes in weight and health behaviours during the transition to higher education and to investigate which health behaviour changes were related to weight change. On average students gained 2.7 kg between the last semester at high school and the beginning of the second year at college or university (time span of 1.5 years), with boys gaining 4.2 kg and girls 1.9 kg. Although a previous study [10] showed that weight gain during the first semester at college or university is smaller in Europe compared to US, weight gain in the present study is comparable to the weight gain (ranging from 1.5 to 4.1 kg) during the first two years at college or university found in US students [14,15,40,41] despite the shorter time span. This indicates that a considerable amount of weight might be gained during the transition period from high school to the start of college or university; a period that was missed in previous studies collecting baseline measurements at the start of the freshman year. Although the amount of weight gain was large in boys and moderate in girls and higher than the average young adults’ weight gain of 0.6-1.0 kg/year [26,42,43], an increase in BMI does not necessarily indicate an increase in health risks associated with overweight. The prevalence of overweight doubled from high school to higher education, but still 84% of students were normal weight at college or university and currently not at increased health risk. However, if the observed weight gain continues across the lifespan, overweight prevalence and associated health risks might increase. Waist circumference is more strongly related with risk of coronary heart diseases than BMI [44]. Only in boys a modest increase in waist circumference was found. The large increase in BMI with modest changes in waist circumference might indicate that part of the weight gain is due to an increase in fat free mass. However, a previous study in European students [10] also found no changes in waist circumference, despite a significant increase in fat percentage and fat mass and no change in fat free mass.

Looking at the energy expenditure side of the energy balance, we found a decrease in sport participation in both boys and girls which was related to weight gain. This might be explained by the fact that fewer college or university students are members of a sports-club compared to high school students [45,46], because they move away from their home town or due to lack of time [47-50]. A decrease was found in some sedentary behaviours (e.g. TV/DVD viewing, playing computer games), while other sedentary behaviours (e.g. internet use, time spent studying) increased. The increase in leisure time internet use was related to weight gain which is in line with previous studies showing a positive association between increased sitting time and weight change in young adults [51,52]. In contrast, increases in sitting time while studying was related to less weight gain. Self-control might be confounding this relationship. High self-control is related with higher levels of physical activity [53], healthier eating patterns [53], less alcohol consumption [54,55], lower BMI [53] and sticking to study schedules [56]. After following a study intervention program practicing self-control, undergraduate students not only studied more and improved study habits, but also improved their behaviour in many ways outside the context of academic habits (such as decrease in tobacco and alcohol consumption and an increase in healthy dietary habits) [57]. So it might be that students who have more self-control to study regularly are also more disciplined to maintain their weight, to eat healthy, control drinking and to set time aside for physical activity [58].

Looking at the energy intake side of the energy balance, consumption of healthy as well as unhealthy foods decreased during the transition from high school to college or university. This general decrease in dietary intake is in contrast with the observed weight gain, but is in line with findings of previous studies [28,59]. Despite the decrease of fruit and vegetable intake in both genders, an increase in fruit and vegetable intake was related to less weight gain, but only in girls. Because fruits and vegetables are high in water and fiber, incorporating them in the diet can reduce energy density, promote satiety and decrease energy intake [60]. Several longitudinal studies have shown that higher fruit and vegetable intake is protective against weight gain [61-63]. However, it is not clear why this association was only found in girls in the present study, and not in boys. A previous 24 year longitudinal study (between the ages 12 to 36 years) also only found low vegetable intake to be related with more weight gain in women [64], while another study in adults only observed an inverse association between fruit and vegetable intake and weight gain in men [65]. Although fruit and vegetable consumption was generally lower in boys compared to girls in the present study, the decrease in fruit and vegetable consumption was similar in both genders. The lack of association between changes in fruit and vegetable consumption and weight gain in boys can therefore not be explained by a smaller decrease in boys. Adequate fruit and vegetable intake is also a marker for a healthy diet [66]. This may indicate that girls having larger fruit and vegetable consumption engaged in healthy behaviours or were dieting using positive weight loss methods.

Despite expectations, no other changes in dietary behaviours were related to weight gain. In a previous US study using a 69-item FFQ, decreased consumption of fruit and vegetables was also the only significant dietary predictor of freshman weight gain [27]. It is possible that other unmeasured dietary behaviours such as recent dieting, meal frequency, meal location (e.g. at restaurant or friend’s place), evening or late-night snacking or skipping breakfast are more important predictors of weight gain [10,42,67-69].

The increase in alcohol consumption only contributed to weight gain in boys. In previous qualitative studies alcohol use was mentioned to contribute to weight gain because of the calories the drinks add, but also because of the unhealthy and excess eating that co-occurs [49,58]. In the study of Lloyd-Richardson et al. [17] nearly half of students reported overeating and making unhealthy food choices following drinking. Heavy drinkers also tend to frequently skip breakfast [70]. The fact that girls in the present study showed a smaller increase in alcohol consumption and also a lower absolute alcohol consumption at both time points compared to boys, might explain why alcohol consumption only contributed to weight gain in boys. Another study also found weight gain to be associated with increased alcohol consumption during the freshman year in boys and with lower baseline fruit and vegetable consumption in girls [28]. As only two previous studies [9,28] examined differences in correlates of weight gain between boys and girls, these gender differences need to be further explored.

The main strength of this study is that this was the first European study that spanned the period from high school to college or university and the first study ever not using retrospective measurements to study this transition period. Another strength is the objective assessment of BMI and waist circumference. Although we used validated questionnaires, a first limitation is that health behaviours were not objectively assessed and might include over- and underestimations. The main study limitation is the high attrition rate. Althought there was no difference in BMI between participants and drop-outs at baseline, it is likely that students who gained more weight were less keen on participating in follow-up measurements, which may have led to an underestimation of the real weight gain. The low explained variance of the regression model indicates that other unmeasured variables (e.g. hours of sleep [26], eating behaviours [10,42,67-69], stress [21]) might explain more variance in weight change during this transition period. Finally, this study was limited to a sample of students in one single city and findings may not be applicable to students studying in other cities or in other cultural contexts.

In summary we can conclude that the transition from high school to college or university is an at risk period for weight gain and unfavourable changes in physical activity, internet use, fruit and vegetable consumption and alcohol consumption in Belgian students. The similar weight gain found in this study compared to US studies spanning a longer period but not including the transition from high school to college or university suggests that a considerable amount of changes happen during this transition period. Interventions to prevent weight gain in college or university students should therefore already start in high school and continue at college or university. Although promoting sports participation and limiting leisure time internet use might be an effective overweight prevention strategy in both genders, intervention programs may need to target boys and girls somewhat differently, with more attention to alcohol consumption in boys and fruit and vegetable intake in girls. Future experimental research needs to confirm the effect of such intervention programs. In order to be able to develop a successful intervention program, future studies need to investigate which individual, psychosocial and environmental factors are related to changes in these health behaviours during this transition period.
